# Breast Cancer Stem Cells and Immunogenicity Profile in High-Risk Early Triple-Negative Breast Cancer: A Pilot Study

**DOI:** 10.3390/ijms26093960

**Published:** 2025-04-22

**Authors:** Ariadna Roqué-Lloveras, Ferran Pérez-Bueno, Xavier Pozo-Ariza, Emma Polonio-Alcalá, Sira Ausellé-Bosch, Glòria Oliveras, Gemma Viñas, Teresa Puig

**Affiliations:** 1 Medical Oncology Department, Catalan Institute of Oncology Girona, 17007 Girona, Spain; aroque@iconcologia.net; 2 Precision Oncology Group (OncoGIR-Pro), Institut d’Investigació Biomèdica de Girona (IDIBGI), 17190 Salt, Spain; epolonio@idibgi.org; 3 New Therapeutic Targets Laboratory (TargetsLab)–Oncology Unit, Medical Science Department, Faculty of Medicine, University of Girona, 17003 Girona, Spain; fperezbueno.girona.ics@gencat.cat (F.P.-B.); sira.auselle@udg.edu (S.A.-B.); goliveras@iconcologia.net (G.O.); 4 Pathological Anatomy Department, Dr. Josep Trueta University Hospital, 17007 Girona, Spain; fjpozoa.girona.ics@gencat.cat

**Keywords:** triple-negative breast cancer, breast cancer stem cells, PD-L1, neoadjuvant chemotherapy, adjuvant chemotherapy, targeted therapy

## Abstract

Triple-negative breast cancer (TNBC) is an aggressive subtype requiring further knowledge of biomarkers to improve targeted therapy. A major resistance mechanism involves breast cancer stem cells (BCSCs) evading the immune system. Neoadjuvant or adjuvant chemotherapy may alter BCSCs and the patients’ immune response. We conducted a retrospective study including 29 early-stage TNBC patients resistant to chemotherapy diagnosed at the Catalan Institute of Oncology (Girona, Spain) in 2010–2019. We obtained 44 paired tumor samples (pre- and post-chemotherapy) from the Tumor Biobank, assessing BCSC biomarkers (CD44, CD24, and ALDH1), PD-L1, and percentages of stromal tumor-infiltrating lymphocytes (TILs). Clinicopathological characteristics were also collected. At baseline, 68% of tumors had high CD44 expression, 55% showed low CD24 expression, 9% had high ALDH1 expression, 91% were PD-L1-negative (<1%), and 64% had a low percentage of stromal TILs. PD-L1 expression significantly increased post-chemotherapy, with 50% of initially negative tumors becoming PD-L1 positive (≥1%) (*p* = 0.006). No significant changes were observed in BCSC markers or TILs. No association was found between baseline BCSCs and increased PD-L1 expression post-chemotherapy. At a median follow-up of 58.9 months, 48.3% of patients were alive, with non-significant favorable trends in time to progression, disease-free survival, and overall survival in the PD-L1 positivization cohort post-chemotherapy. In conclusion, high-risk early-stage TNBC tumors increased PD-L1 expression after chemotherapy, potentially affecting clinical outcomes. BCSCs remained stable and independent of the tumor immunogenicity post-chemotherapy. Further studies are needed to explore the relationship between BCSCs and the immunogenicity profile, for development of new combined therapeutic strategies.

## 1. Introduction

Breast cancer is the most commonly diagnosed cancer globally and the fifth leading cause of cancer-related deaths, with an estimated 2.3 million new cases and 685,000 deaths in 2020 [1]. Triple-negative breast cancer (TNBC), a subtype defined by the absence of expression of estrogen receptor (ER), progesterone receptor (PR), and human epidermal growth factor receptor 2 (HER2), accounts for 10–20% of all breast cancers. TNBC is among the most lethal subtypes, mainly due to its aggressive nature and the lack of targeted therapies, leaving chemotherapy (CT) as the cornerstone of standard treatment [2].

Significant efforts are currently being made to develop new targeted therapies for this aggressive subtype, due to the high rates of tumor recurrence and metastasis [3]. In breast cancer, a major factor contributing to this tumoral persistence is the presence of a small population of tumorigenic cells within tumors known as breast cancer stem cells (BCSCs). These cells are resistant to conventional treatments, such as CT and radiotherapy, and exhibit several stem cell-like properties, including self-renewal and pluripotency [4]. BCSCs also play a key role in the epithelial–mesenchymal transition (EMT), a process that enables cancer cell migration and formation of metastasis [5]. Additionally, BCSCs can be identified by specific surface markers, including high expression of CD44 (CD44^+^) and low or non-expression of CD24 (CD24^−/low^) [4]. Another feature of BCSC is the high level of aldehyde dehydrogenase 1 (ALDH1), an enzyme that contributes to cell detoxification by oxidizing aldehydes [6]. Since TNBC is an aggressive subtype within all breast cancer tumors and is characterized by typical traits of BCSCs, such as high relapse rates and metastatic development, our research aims to explore whether targeting BCSCs with individual or combination therapies offers potential new treatment strategies for TNBC [7].

Over the past decade, immunotherapy has revolutionized the treatment landscape for various cancers. Its application is now showing promise in the TNBC subtype, in the context of the latter’s greater genomic instability, higher mutation rate, and increased neoantigen production, which collectively result in heightened immunogenicity [8]. Immune checkpoint inhibitors (ICIs) have shown effectiveness in treating both early-stage and metastatic TNBC, with the potential to significantly improve patient survival outcomes [9,10,11], particularly in those with high expression of programmed death-ligand 1 (PD-L1) [11,12]. However, resistance to these therapies can arise from the immunosuppressive microenvironment fostered by BCSCs. This malignant subpopulation can evade the immune system and express high levels of PD-L1, making them potential targets for ICIs, either as monotherapy or in combination with other treatments to enhance therapeutic responses [13]. Additionally, the presence of tumor-infiltrating lymphocytes (TILs) within the tumor and surrounding stroma has been associated with improved treatment response and survival in TNBC patients [14,15]. Thus, it is essential to better characterize the BCSC population in TNBC to develop more effective therapies targeting both immune response and BCSCs.

Therefore, the primary aim of this study was to analyze changes in BCSCs and the immunogenic profile in paired tumor samples from TNBC patients treated with neoadjuvant or adjuvant CT. Specifically, this study aimed to evaluate the impact of CT on the BCSC subpopulation and the overall immunogenic profile.

## 2. Results

### 2.1. Clinicopathological Characteristics

From 2010 through 2019, a total of 29 patients diagnosed with TNBC were retrospectively included in our study. The clinicopathological variables are summarized in Table 1. The mean age of the patients was 51 years. Of the tumors, 79.3% were invasive ductal carcinomas, while 75.9% were grade III, and the mean Ki-67 index, reflecting tumor proliferation, was high at 57.5%. According to the eighth edition of the AJCC-TNM classification, tumors were most commonly classified as T2 (44.8%) and N1 (41.4%).

Most patients underwent conservative breast surgery (65.5%) and received anthracyclines and taxanes as CT (79.4%). Additionally, 75.9% of the patients underwent adjuvant radiotherapy. Germline mutations in *BRCA1* or *BRCA2* were present in four patients (13.8%).

At the data cutoff on 19 January 2024, with a mean (median) follow-up of 62.67 (58.9) months, 48.3% of the patients were alive. Relapses occurred in 44.8% of the patients, predominantly as visceral metastasis (92.3%). Considering only the biomarker-analyzed cohort, the median disease-free survival (DFS) was 25.5 months (95% confidence interval (CI), 12.8 − not achieved (NA)), the median overall survival (OS) was 35.7 months (95% CI, 22.7 − NA), and the median time to progression (TTP) was 29.7 months (95% CI, 12.8 − NA) (Figures S1–S3).

### 2.2. Basal Expression of BCSCs and Immunogenic Biomarkers

**Figure 1:**
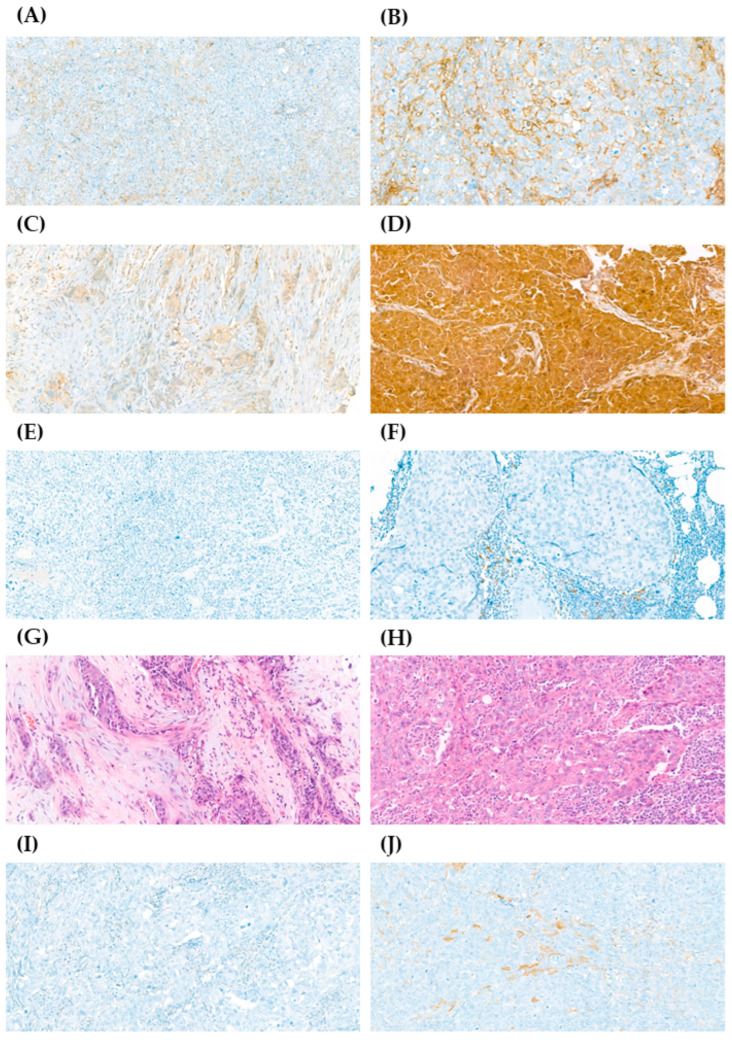
Immunohistochemistry (IHC) staining of CD44, CD24, PD-L1, tumor-infiltrating lymphocytes (TILs), and ALDH1 in samples from patients with early-stage triple-negative breast cancer (TNBC). The expression of CD44, CD24, and ALDH1 was graded as 0+, 1+, 2+, or 3+; PD-L1 expression was graded as negative < 1% or positive ≥ 1%; the percentage of stromal TILs was graded as 0–10%, 10–40%, or 40–90%. (**A**) Representative image from sample with low expression of CD44 (1+) (40×). (**B**) Representative image from sample with high expression of CD44 (3+) (40×). (**C**) Representative image from sample with low expression of CD24 (1+) (40×). (**D**) Representative image from sample with high expression of CD24 (3+) (40×). (**E**) Representative image from sample with negative expression of PD-L1 (20×). (**F**) Representative image from sample with positive expression of PD-L1 (40×). (**G**) Representative image from sample with 0–10% of stromal TILs (40×). (**H**) Representative image from sample with 40–90% of stromal TILs (40×). (**I**) Representative image from sample with no expression of ALDH1 (0+) (40×). (**J**) Representative image from sample with high expression of ALDH1 (2+) (40×).

From the 29 selected TNBC patients, tumor samples from 22 were available for biomarker analysis including BCSC markers (CD44, CD24, ALDH1) and immunogenic markers (PD-L1, and TILs) (Figure 1). Differential expression of CD44^+^/CD24^−^ and elevated ALDH1 expression are directly associated with BCSCs [4,6]. Biomarker analysis in samples acquired before CT revealed that 68% of patients exhibited high CD44 expression and 55% demonstrated low CD24 expression. Interestingly, 41% of patients exhibited both high CD44 and low CD24 basal expression (CD44^+^/CD24^−/low^), but this association was not statistically significant (*p* = 0.227) (Figure S4A). Only 9% of patients showed high ALDH1 basal expression. Additionally, 50% of patients demonstrated both low CD24 and low ALDH1 basal expression (*p* = 0.714) (Figure S4B), while 64% had both low ALDH1 and high CD44 basal expression (*p* = 0.805) (Figure S4C).

Regarding the immunogenic profile of this chemoresistant TNBC population, only 9% of patients had baseline PD-L1 expression ≥ 1%, whereas low basal percentage of stromal TILs was observed in 64% of patients. About 64% of patients showed both low basal percentage of stromal TILs and PD-L1 expression < 1%, demonstrating a statistically significant association (*p* = 0.030) (Figure S4D). This relationship persisted in the post-CT samples, with 41% of patients exhibiting both PD-L1 < 1% and a low percentage of stromal TILs, while 36% of patients had a moderate/high percentage of stromal TILs and PD-L1 expression ≥ 1% (*p* = 0.015) (Figure S4E).

Details of the descriptive biomarker analysis are provided in Table S1.

### 2.3. Effect of CT on BCSCs and Immunogenic Biomarkers

The paired tumor samples from the 22 chemoresistant TNBC patients included specimens collected both before and after CT. The McNemar test was applied to assess the differences between pre- and post-treatment samples.

**Figure 2:**
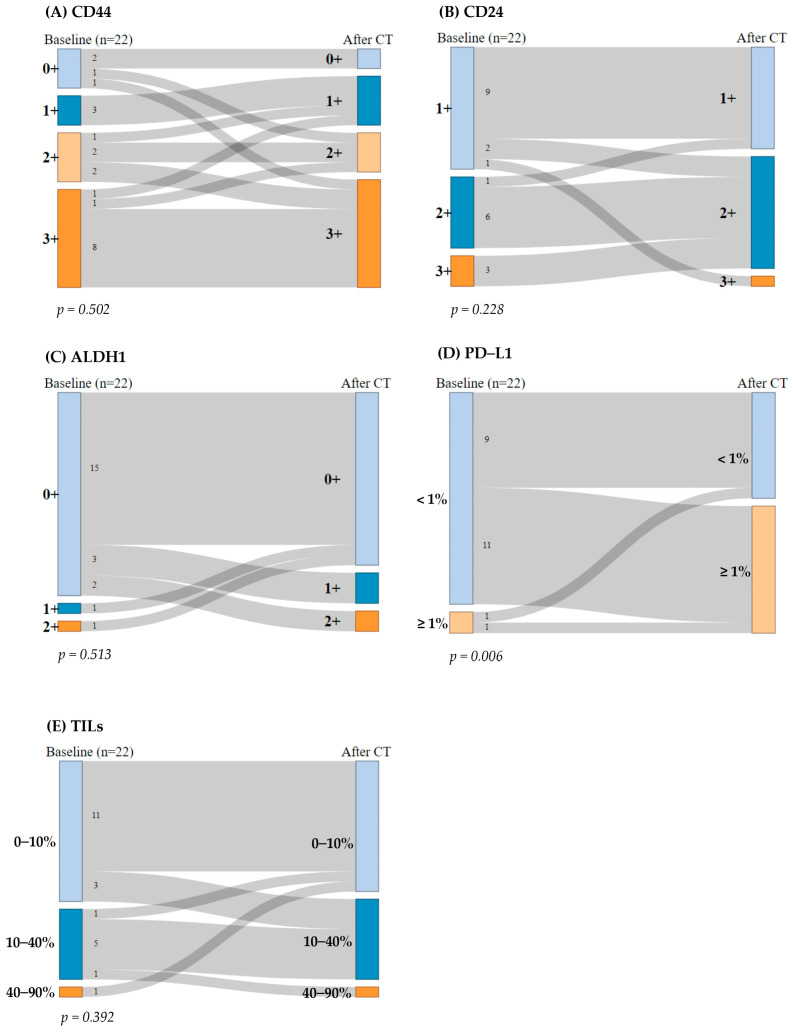
Sankey diagram: breast cancer stem cells (BCSCs) and immunogenicity biomarkers before and after chemotherapy (CT). The McNemar–Bowker test for paired data was performed. (**A**) CD44 expression, (**B**) CD24 expression, (**C**) ALDH1 expression, (**D**) programmed death-ligand 1 (PD-L1) expression, and (**E**) percentage of stromal tumor-infiltrating lymphocytes (TILs).

Regarding CD44, 13 patients (59%) consistently exhibited high CD44 expression (2+ or 3+), while 5 patients (23%) displayed low expression (0+ or 1+) before and after CT. Additionally, two (9%) patients showed an increase in CD44 expression after CT, whereas two (9%) patients experienced a decrease in CD44 expression. The McNemar test indicated no significant differences in CD44 expression pre- and post-CT (*p* = 0.502) (Figure 2A).

Regarding CD24, nine patients (41%) maintained CD24 expression at 1+ and six patients (27%) at 2+ before and after CT. Four patients (18%) exhibited a reduction in CD24 expression by one grade after CT, while only one patient (4.5%) showed an increase in CD24 expression (from 1+ to 3+). No samples were categorized as 0+ expression. The McNemar test revealed no significant differences in CD24 expression before and after treatment (*p* = 0.228) (Figure 2B).

Regarding ALDH1, 15 patients (68%) had low ALDH1 expression (0+) before and after CT. After the treatment, five patients (23%) showed an increase in ALDH1 expression, while two patients (9%) with previously positive ALDH1 expression experienced a reduction in its expression after CT. The McNemar test indicated no significant change in ALDH1 expression pre- and post-CT (*p* = 0.513) (Figure 2C).

Regarding PD-L1, nine patients (41%) consistently exhibited PD-L1 expression < 1%, while only one patient (4.5%) displayed PD-L1 expression ≥ 1% before and after CT. Interestingly, 11 patients (50%) transitioned from PD-L1 negative (<1%) to PD-L1 positive (≥1%) after CT, which was statistically significant (*p* = 0.006). Thus, the McNemar test indicated statistically significant differences in PD-L1 expression pre- and post-CT (*p* = 0.006) (Figure 2D).

With regard to TILs, 11 patients (50%) maintained their percentage of stromal TILs at 0–10% and five patients (23%) at 10–40% before and after CT. Four patients (18%) exhibited an increase in their percentage levels of stromal TILs by one grade after CT, while two patients (9%) showed a decrease in percentage of stromal TILs. The McNemar test revealed no significant differences in percentages of stromal TILs before and after treatment (*p* = 0.392) (Figure 2E).

### 2.4. Effect of PD-L1 Positivization on Clinical Outcomes

The impact of PD-L1 positivization on clinical outcomes was assessed among patients whose tumors became PD-L1 positive after CT. According to Fisher’s exact test, patients who became PD-L1 positive after CT did not show greater enrichment of the BCSC population at baseline, since there were no significant differences compared with those patients who remained PD-L1 negative (CD44, *p* = 0.423; CD24, *p* = 0.725; ALDH1, *p* = 1.000). Hence, these findings indicate that tumors that became PD-L1 positive did not exhibit a larger BCSC population (Supplementary Tables S2–S4).

**Figure 3:**
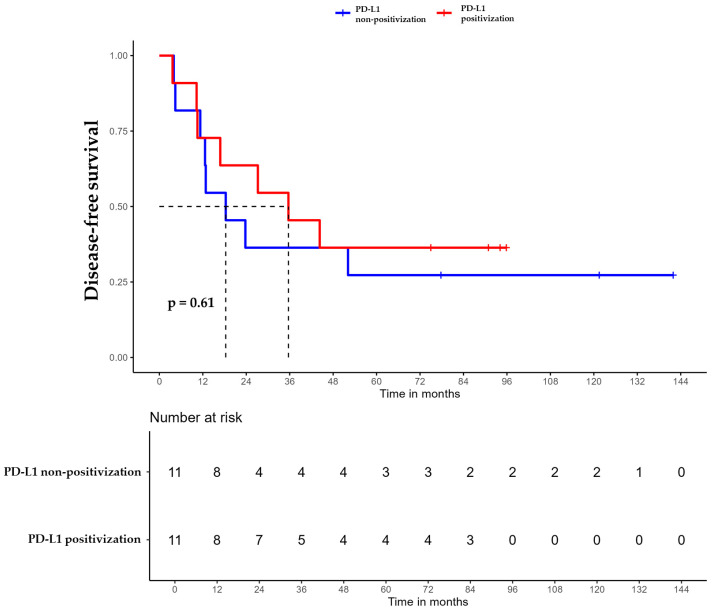
Disease-free survival (DFS) of triple-negative breast cancer (TNBC) patients with positivized PD-L1 after chemotherapy (red) compared with patients with non-positivized PD-L1 expression (blue).

**Figure 4:**
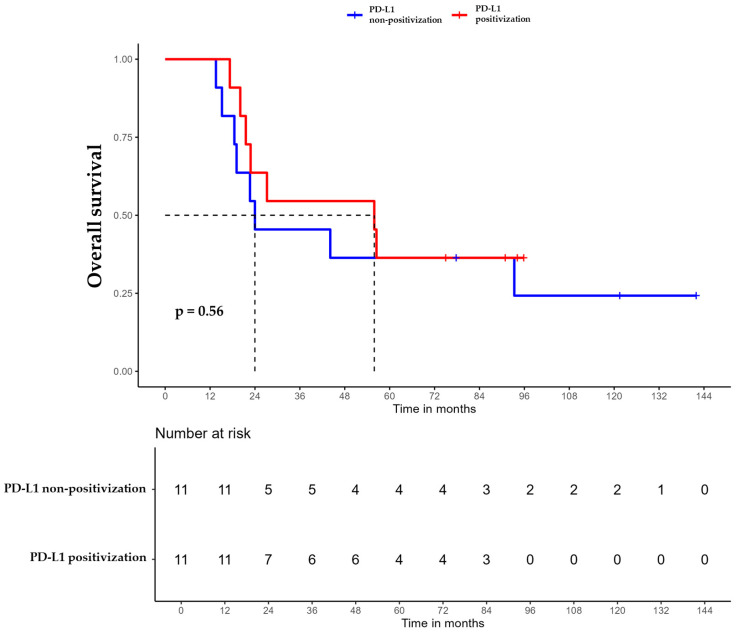
Overall survival (OS) of triple-negative breast cancer (TNBC) patients with positivized PD-L1 after chemotherapy (red) compared with patients with non-positivized PD-L1 expression (blue).

**Figure 5:**
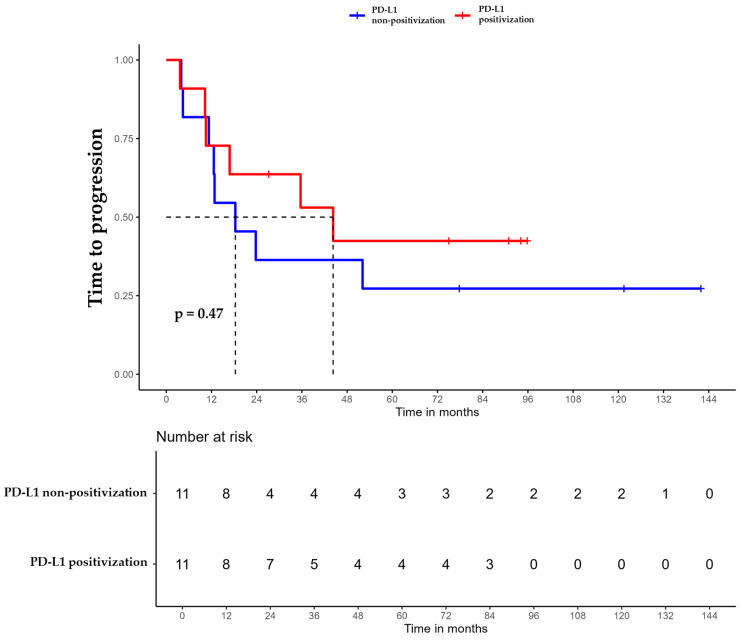
Time to progression (TTP) of triple-negative breast cancer (TNBC) patients with positivized PD-L1 after chemotherapy (red) compared with patients with non-positivized PD-L1 expression (blue).

For patients whose tumors changed to PD-L1 positive status post-CT, the median DFS was 35.6 months (95% CI, 16.8 NA) (Figure 3), the median OS was 55.9 months (95% CI, 22.8 NA) (Figure 4), and the median TTP was 44.3 months (95% CI, 16.8 NA) (Figure 5). In contrast, for the PD-L1-non-positivization cohort after CT, the median DFS was 18.3 months (95% CI, 12.6 NA), the median OS was 24 months (95% CI, 19.1 NA), and the median TTP was 18.3 months (95% CI, 12.6 NA). However, the differences observed between the two cohorts did not reach statistical significance (DFS, *p* = 0.61; OS, *p* = 0.56; and TTP, *p* = 0.47).

## 3. Discussion

In the past decade, research on biomarkers in TNBC has focused on identifying molecules with prognostic and predictive capabilities to improve patient stratification and guide therapeutic decisions. The BCSC subpopulation has been suggested as a potential target for breast cancer treatment, either using stem cells as cellular vehicles for targeted delivery therapy or through BCSC-targeted therapies [7].

The clinicopathological characteristics of the TNBC patients in our study population align with the aggressiveness widely described in the literature for this subtype of breast cancer [2]: young women with locally advanced tumors and poor prognostic factors, such as lymph node involvement, high nuclear grade tumors, and high Ki-67 indices. Our biomarker study population was by definition chemoresistant, since none of the patients achieved a pathological complete response (pCR) after CT in a curative setting or they developed metastasis after adjuvant CT. Cortazar et al. described differences between responders and non-responders in terms of survival outcomes [16]. In line with those previous findings, our chemoresistant cohort exhibited aggressive progression and poor outcomes, with more than 50% of the patients succumbing to the disease despite early-stage breast cancer diagnosis. It is precisely in this high-risk TNBC population that a more thorough search for biomarkers is needed to improve outcomes.

To our knowledge, this is the first study to analyze BCSC behavior in chemoresistant TNBC patients before and after standard CT based on anthracyclines and taxanes. Most of the literature about BCSC biomarkers is derived from in vitro assays using breast cancer cell lines and xenografts [17]. In vivo studies often evaluate the prognostic capacity of BCSCs by comparing responders and non-responders to CT, rather than assessing BCSC behavior during treatment [18]. The presence of residual BCSCs in the tumor microenvironment after CT could explain the chemoresistance of our non-pCR cohort, suggesting a potential druggable target.

In our analysis, we observed that the chemo-naïve basal tumor samples had high CD44 and low CD24 expression (CD44^+^/CD24^−/low^ phenotype), which could be interpreted as BCSC-enriched, consistent with previous studies on chemoresistant cohorts [18]. Unexpectedly, we detected low basal expression of ALDH1, although contradictory to some previous studies, this may be attributed to several factors including tumor heterogeneity in TNBC, methodological differences in ALDH1 detection, and the influence of the tumor microenvironment, particularly TILs. Specific TNBC subtypes might inherently express lower levels of ALDH1, contributing to this variability [19]. It should be highlighted that our study did not measure the functional protein activity of ALDH1 and consequently, the role of ALDH1 in chemoresistance could potentially have been underestimated.

Interestingly, the BCSC subpopulation in our cohort remained stable after CT and no significant differences were found. A slight upward trend in BCSC population after treatment was observed, which could be confirmed in future analysis with a larger sample size. However, detecting changes in a baseline population that is already enriched in BCSCs is challenging. In line with our findings, Resetkova et al. reported no ALDH1 expression increase in the post-neoadjuvant CT sample from a non-selected breast cancer cohort [20].

Regarding the immunogenic profile, most samples had low percentages of stromal TILs at baseline, consistent with the low response to CT in this high-risk cohort. Various studies have demonstrated TILs as predictors for neoadjuvant response in breast cancer [21]. In our cohort, the percentage of stromal TILs remained low even after CT treatment.

To our knowledge, this is the first study to investigate the potential prognostic role of PD-L1 expression in early-stage TNBC by using tumor samples collected before and after CT. Our results revealed a statistically significant increase in PD-L1 expression post-CT, supporting the hypothesis that anthracycline-based CT could potentially enhance the immunogenicity of breast cancer, making tumors more targetable via immunotherapy [22]. The upregulation of PD-L1 in TNBC following anthracycline/taxane-based CT probably stems from dual, overlapping mechanisms. Firstly, CT directly induces tumor cell stress and DNA damage, triggering pro-inflammatory cytokines such as interferon (IFN)-γ that activate the JAK/STAT pathway, which is a key driver of PD-L1 expression, as an adaptive immune evasion strategy. Concurrently, damaged tumor cells release antigens and activate STING signaling, further amplifying PD-L1 [23]. Secondly, CT remodels the tumor microenvironment; infiltrating CD8+ T cells and macrophages also secrete IFN-γ [24], while partial tumor regression may expose preexisting PD-L1-rich immune niches previously masked by high cellularity. Residual tumors also exhibit genomic instability (e.g., TP53 mutations) and clonal selection, favoring PD-L1 positive subpopulations resistant to therapy [25].

Clinically, this adaptive PD-L1 induction reflects a paradox; while associated with aggressive residual disease and poor prognosis, it may simultaneously prime tumors for immunotherapy [23]. The interplay between the immunogenic effects of CT (e.g., antigen release) and the tumor’s counteractive PD-L1-driven immunosuppression underscores the rationale for combining CT with immune checkpoint inhibitors in TNBC.

These findings suggest that CT-mediated PD-L1 upregulation in TNBC could potentially enhance responsiveness to CT and immunotherapy combinations, as evidenced by improved pCR rates in the KEYNOTE-522 trial, though further studies are needed to confirm this biological relationship [10]. However, it is important to consider potential differences in PD-L1 assessment depending on the antibody clones or scoring systems used, as well as the inherent subjectivity in the interpretation of IHC results. Further investigations are also needed to evaluate the potential role of escalating immunotherapy or de-escalating CT after PD-L1 positivization in this TNBC population with low basal PD-L1 expression.

Interestingly, as expected and previously described, we found a significant association between both immunogenicity biomarkers, PD-L1 and TILs, in pre- and post-CT samples [26]. However, no statistically significant differences were found between PD-L1 positivization and the expression of BCSC biomarkers at baseline. A study by Guevara M et al. revealed that CD56+ natural killer cells reduced CD133+ populations (a CSC marker) by 40% in non-small cell lung cancer. That study demonstrated an inverse correlation between the density of these NK cells and tumor metastatic capacity, confirming a less immunogenic environment in CSC-enriched tumors [27]. In contrast, Almozyan et al. demonstrated that PD-L1 promoted OCT4 and Nanog expression in BCSCs by sustaining PI3K/AKT pathway activation [28]. Consequently, the current authors believe that the interaction between immune and BCSC markers requires further investigation.

Additionally, our findings revealed that the PD-L1 positivization cohort exhibited higher DFS, OS, and TTP compared to the PD-L1 non-positivization cohort, although the differences did not reach statistical significance. The prognostic value of PD-L1 expression by IHC in breast cancer has been suggested by discordant results across several studies, due to different antibody clones, cutoff points, and scoring systems. While few studies demonstrated a strong correlation between PD-L1 expression and clinical outcomes, some identified PD-L1 as a biomarker for lower survival rates, or no association was found. AiErken et al. observed that PD-L1-positive patients showed significantly greater DFS and OS compared with PD-L1-negative patients, using tumor samples from resection surgical specimens without neoadjuvant treatment [29]. However, Muenst et al. observed in univariate analysis that PD-L1 was associated with worse OS; PD-L1 expression was quantified using the modified histoscore (H-score) calculated through semi-quantitative assessment of both the intensity of staining and the percentage of PD-L1-positive tumor and immune cells [30]. To our knowledge, our study is the first to evaluate changes in PD-L1 expression after CT in TNBC and their impact on survival. Existing evidence focuses on baseline PD-L1 expression as a predictive biomarker. However, the dynamic modulation of PD-L1 post-treatment and its prognostic significance have not been previously explored, underscoring the novelty of our findings. Further research is needed to confirm the prognostic role of the PD-L1 expression changes in this group of patients with such poor prognosis.

Our study included certain methodological limitations that could have impacted the findings. Firstly, it was a retrospective analysis, which inherently carries potential biases. Secondly, the number of samples with adequate tissue for IHC analysis was smaller than anticipated. As this was a pilot study with only 29 patients, the results may be limited in terms of generalizability and statistical power, and multivariate analysis could not be conducted due to insufficient statistical strength. The small sample size could also have prevented the detection of certain associations or significant differences in biomarker expression and clinical outcomes. Thirdly, some of the tumor samples were relatively aged, which may have affected the IHC outcomes. Moreover, the use of different antibody clones could have led to variations in the assessment of biomarker expression, potentially leading to differing results. It should be noted that for biomarkers routinely assessed in clinical practice, such as PD-L1, we employed the same antibody that is used in routine diagnostics at our center. Lastly, comparison between our cohort and patients with good prognosis was not performed, due to the lack of viable tumor samples after CT, making it impossible to evaluate biomarkers in pCR patients. Nonetheless, in this regard, the baseline-enriched BCSCs and the low PD-L1 values and percentages of stromal TILs detected in our baseline samples were comparable to expectations for this poor prognostic subgroup.

## 4. Materials and Methods

### 4.1. Study Design

This study was approved by the Institutional Ethics Committee. This retrospective pre-post study included 29 patients diagnosed with early TNBC (defined as estrogen receptor < 10%, progesterone receptor < 10%, and HER2 negative 0 or 1+, as used in clinical practice) at the Catalan Institute of Oncology of Girona between 2010 and 2019. Clinicopathological variables and tumor characteristics were retrospectively collected from patients’ clinical records. Patient identifiers were coded to ensure anonymity. Data from patients were obtained from the Hospital Cancer Registry database.

### 4.2. Immunohistochemistry (IHC) Assay of Paired Tumor Samples

Selected patients provided two formalin-fixed paraffin-embedded (FFPE) samples: one from pre-treated core biopsy or surgical specimen and another from the surgical specimen after neoadjuvant CT with anthracyclines and taxanes or from metastatic tissue after adjuvant CT. An experienced pathologist determined the best representative area of each tumor sample. Samples were obtained from the Girona Biomedical Research Institute (IDIBGI) Tumor Biobank after patients signed informed consent forms. Biomarker analysis was performed on samples from 22 patients (18 patients who received neoadjuvant CT and did not achieve pCR and 4 patients who received adjuvant CT; in total, 44 paired samples). We excluded 7 patients due to insufficient pathological samples for IHC and hematoxylin-eosin (H-E) staining, after pathologist evaluation.

BCSC biomarkers (CD44, CD24, and ALDH1) and PD-L1 were evaluated via IHC using an automatic VENTANA BenchMark ULTRA machine (Roche, Ventana Medical Systems, Tucson, AZ, USA). Antibodies used included CD24 (ALB9, Abcam, Cambridge, UK), CD44 (SP37, Roche), ALDH1 (EP1933Y for detecting ALDH1A1 isoform, Abcam), and PD-L1 (SP142, Roche), with dilutions adjusted according to manufacturer’s instructions. TILs were evaluated via H-E staining using an automatic VENTANA HE 600 machine (Roche). Two experienced pathologists conducted interpretation of IHC and H-E staining using optical microscopy. In accordance with the literature review, membrane CD44 staining, cytoplasmic and circumferential membranous CD24 staining, cytoplasmic ALDH1 staining, and membranous, cytoplasmic, and inflammatory infiltrate PD-L1 staining were assessed. BCSC biomarkers were qualitatively evaluated and categorized into four groups. PD-L1 expression was semi-quantitatively assessed as the percentage of tumor cells showing positive staining (≥1% defined as positive, following literature and routine clinical practice). TILs were evaluated according to the International TILs Working Group 2014 recommendations, graded as a percentage of stromal cell areas [14]. The biomarker assessment is summarized in Table 2.

### 4.3. Statistical Analysis

Clinicopathological variables were summarized using absolute and relative frequencies for categorical variables, and either mean ± standard deviation or median and interquartile range for numerical variables, depending on data normality. Normality was assessed using the Shapiro–Wilk test. Changes in BCSC and immunogenicity biomarkers due to CT were analyzed using the McNemar–Bowker symmetry test. OS, DFS, and TTP were calculated using the Kaplan–Meier method. The significance level was set at 0.05. Data analysis was performed using IBM SPSS software (version 23.0; SPSS Inc., Chicago, IL, USA) and R (version 4.3.0; The R Foundation, Vienna, Austria).

## 5. Conclusions

Understanding therapeutic resistance remains a major challenge with regard to TNBC. Our study suggests that chemoresistant TNBC tumors with low PD-L1 expression (poorly immunogenic) may have the potential to transition toward higher PD-L1 expression (more immunogenic) following CT. This potential conversion could allow the adjustment of therapeutic strategies that combine or sequence CT and immunotherapy in this high-risk TNBC patient population. In addition, we observed a BCSC-enriched population and a slight trend toward upregulation after CT. However, further research is required to validate these findings and to better understand the relationship between BCSCs and the immunogenic profile of these tumors, as well as how these insights could be translated into clinical applications.

## Figures and Tables

**Table 1:** Clinicopathological characteristics at baseline.

Variables		Results
Age, mean (range) [median] years		51 (31–81) [47]
Breast cancer histology, *n* (%)	Invasive ductal carcinoma	23 (79.3)
	Invasive lobular carcinoma	1 (3.4)
	Metaplastic carcinoma	3 (10.3)
	Others	2 (6.9)
Histology grade, *n* (%)	I	0 (0)
	II	4 (13.8)
	III	22 (75.9)
	Unknown	3 (10.3)
Estrogen receptor, *n* (%)	0%	28 (96.6)
	1–10%	1 (3.4)
Progesterone receptor, *n* (%)	0%	29 (100)
p53 expression, *n* (%)	0	5 (17.2)
	<25%	3 (10.3)
	>75%	5 (17.2)
	100%	1 (3.4)
	Unknown	15 (51.7)
Ki-67 index, mean (SD) (%)		57.5 (30.7)
Cadherin expression, *n* (%)	Partially positive	1 (3.4)
	Positive	26 (89.7)
	Unknown	2 (6.9)
CK 5/6 expression, *n* (%)	Negative	1 (3.4)
	<10%	3 (10.3)
	11–25%	2 (6.9)
	Unknown	23 (79.3)
HER2 expression, *n* (%)	0	14 (48.3)
	1+	9 (31.0)
	2+	5 (17.2)
	Unknown	1 (3.4)
Breast cancer stage group (TNM ^1^), *n* (%)	IA	1 (3.4)
	IIA	5 (17.2)
	IIB	10 (34.5)
	IIIA	6 (20.7)
	IIIB	4 (13.8)
	IIIC	3 (10.3)
Tumor size, mean (SD) mm		26.02 (26)
Nodal involvement, *n* (%)	N0	11 (37.9)
	N1	12 (41.4)
	N2	3 (10.3)
	N3	3 (10.3)
Surgery type, *n* (%)	Breast conservative surgery	19 (65.5)
	Mastectomy	10 (34.5)
Chemotherapy regimen, *n* (%)	Anthracyclines	2 (6.9)
	Anthracyclines and taxanes	23 (79.4)
	CMF	1 (3.4)
	Others (clinical trial)	2 (6.9)
	None	1 (3.4)
Chemotherapy type, *n* (%)	Neoadjuvant	25 (86.2)
	Adjuvant	4 (13.8)
Radiotherapy, *n* (%)	Yes	22 (75.9)
	No	7 (24.1)
Vital status, *n* (%)	Alive	14 (48.3)
	Deceased	15 (51.7)
Relapse, *n* (%)	Yes	13 (44.8)
	No	16 (55.2)
Relapse localization, *n* (%)	Visceral	12 (92.3)
	Non-visceral	1 (7.7)
*BRCA* germinal status, *n* (%)	Mutated	4 (13.8)
	Non-mutated	15 (51.7)
	Unknown	10 (34.5)

Abbreviations: CMF, cyclophosphamide–methotrexate–fluorouracil. ^1^ TNM: 8th edition of the AJCC-TNM classification.

**Table 2:** Biomarker assessment. Criteria for evaluation of breast cancer stem cell (BCSC) biomarkers (CD44, CD24, and ALDH1), PD-L1, and tumor-infiltrating lymphocytes (TILs).

Biomarker		Expression Evaluation	
BCSCs	CD44, CD24 and ALDH1	0	Total absence
		1+	Mild
		2+	Moderate
		3+	Intense
Immunogenicity	PD-L1	<1%	Negative
		≥1%	Positive
	TILs	According to International TILs Working Group 2014 [14]	

## Data Availability

All data needed to evaluate the conclusions in this paper are present in the text. The datasets used and/or analyzed during the current study are available from the corresponding author on reasonable request. A signed data use agreement and institutional review board approval will be required before the release of research data.

## Supplementary Materials

The following supporting information can be downloaded at https://www.mdpi.com/article/10.3390/ijms26093960/s1.

## Abbreviations

The following abbreviations are used in this manuscript:

ALDH1	Aldehyde dehydrogenase 1 enzyme
BCSC	Breast cancer stem cell
CT	Chemotherapy
DFS	Disease-free survival
EMT	Epithelial-mesenchymal transition
FFPE	Formalin-fixed paraffin-embedded
HER2	Human epidermal growth factor receptor 2
ICI	Immune checkpoint inhibitor
INF-γ	Interferon-γ
IDIBGI	Girona Biomedical Research Institute
IHC	Immunohistochemistry
NA	Not achieved
OS	Overall survival
pCR	Pathological complete response
PD-L1	Programmed death-ligand 1
TIL	Tumor-infiltrating lymphocyte
TNBC	Triple-negative breast cancer
TTP	Time to progression
