# Supplementary material for: Breast Cancer Stem Cells and Immunogenicity Profile in High-Risk Early Triple-Negative Breast Cancer: A Pilot Study
Source: Int J Mol Sci. 2025 Apr 22;26(9):3960. doi: 10.3390/ijms26093960 (PMC12071224; doi:10.3390/ijms26093960)
Supplement: Supplementary file 1 [file ijms-26-03960-s001.zip › ijms-3548479-supplementary.pdf]

SUPPLEMENTARY MATERIAL

Table S1. Biomarker descriptive analysis. CT: Chemotherapy.

**CD44 before CT**, n (%)	0	4 (18)
	1+	3 (14)
	2+	5 (23)
	3+	10 (45)
**CD44 after CT,** n (%)	0	2 (9)
	1+	5 (23)
	2+	4 (18)
	3+	11 (50)
**CD24 before CT,** n (%)	0	0 (0)
	1+	12 (54)
	2+	7 (32)
	3+	3 (14)
**CD24 after CT,** n (%)	0	0 (0)
	1+	10 (45)
	2+	11 (50)
	3+	1 (5)
**ALDH1 before CT**, n (%)	0	20 (91)
	1+	1 (5)
	2+	1 (5)
	3+	0 (0)
**ALDH1 after CT,** n (%)	0	17 (77)
	1+	3 (14)
	2+	2 (9)
	3+	0 (0)
**PD-L1 status before CT**, n (%)	Negative	20 (91)
	Positive	2 (9)
**PD-L1 status after CT**, n (%)	Negative	10 (45)
	Positive	12 (54)
**TILs before CT,** n (%)	0-10%	14 (64)
	10-40%	7 (32)
	40-90%	1 (5)
**TILs after CT,** n (%)	0-10%	13 (59)
	10-40%	8 (36)
	40-90%	1 (5)

Table S2. Cross tabulation of PD-L1 changing patients and CD44 pre-CT expression.

		CD44 expression				Total
		0	1+	2+	3+	
PD-L1 changing patients	No	1 (5%)	2 (9%)	4 (18%)	4 (18%)	11 (50%)
	Yes	3 (14%)	1 (5%)	1 (5%)	6 (27%)	11 (50%)
Total		4 (18%)	3 (14%)	5 (23%)	10 (45%)	22 (100%)

p=0.423

Table S3. Cross tabulation of PD-L1 changing patients and CD24 pre-CT expression.

		CD24 expression			Total
		1+	2+	3+	
PD-L1 changing patients	No	5 (23%)	4 (18%)	2 (9%)	11 (50%)
	Yes	7 (32%)	3 (14%)	1 (4%)	11 (50%)
Total		12 (54%)	7 (32%)	3 (14%)	22 (100%)

p=0.725

Table S4. Cross tabulation of PD-L1 changing patients and ALDH1 pre-CT expression.

		ALDH1 expression			Total
		0	1+	2+	
PD-L1 changing patients	No	10 (45%)	0 (0%)	1 (5%)	11 (50%)
	Yes	10 (45%)	1 (5%)	0 (0%)	11 (50%)
Total		20 (91%)	1 (5%)	1 (5%)	22 (100%)

p=1.000

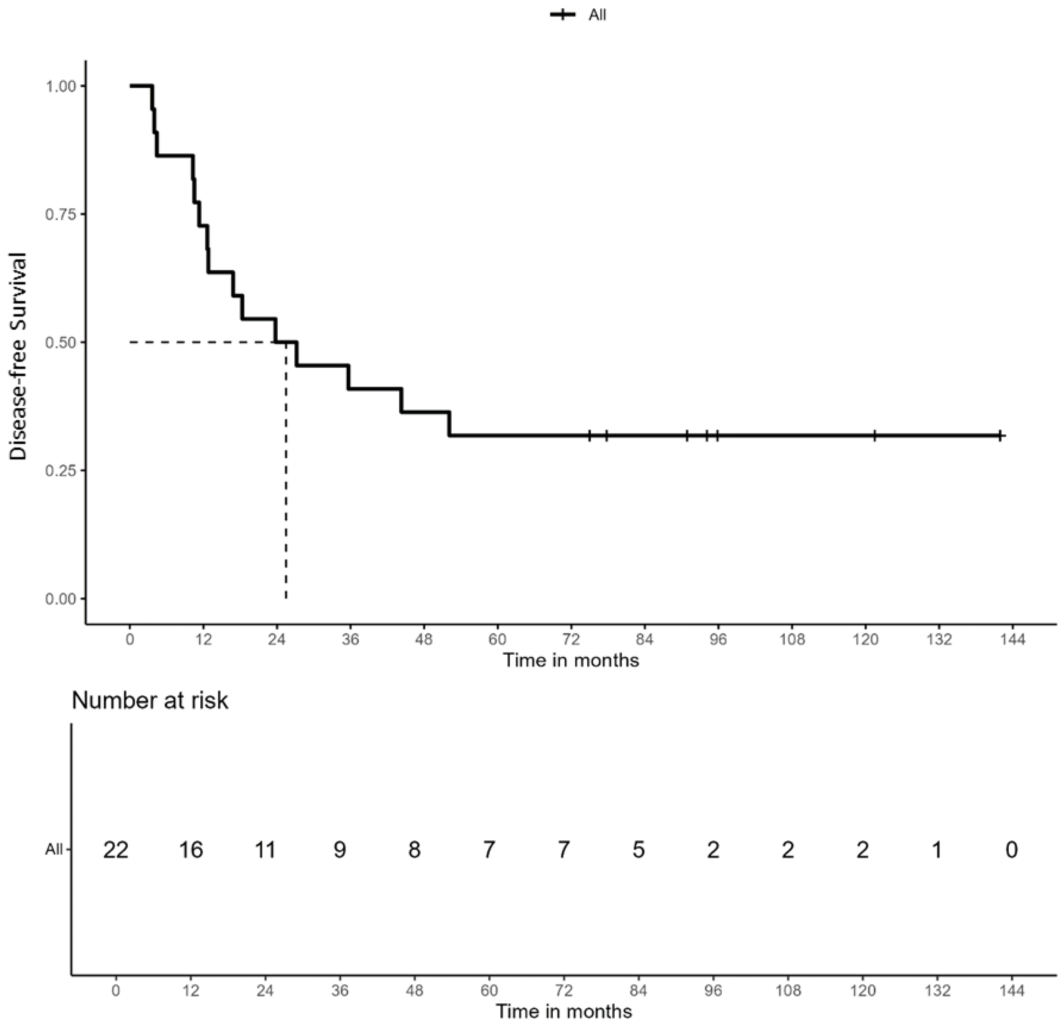

Figure S1. Disease-free survival (DFS) in overall biomarker-assessed-cohort.

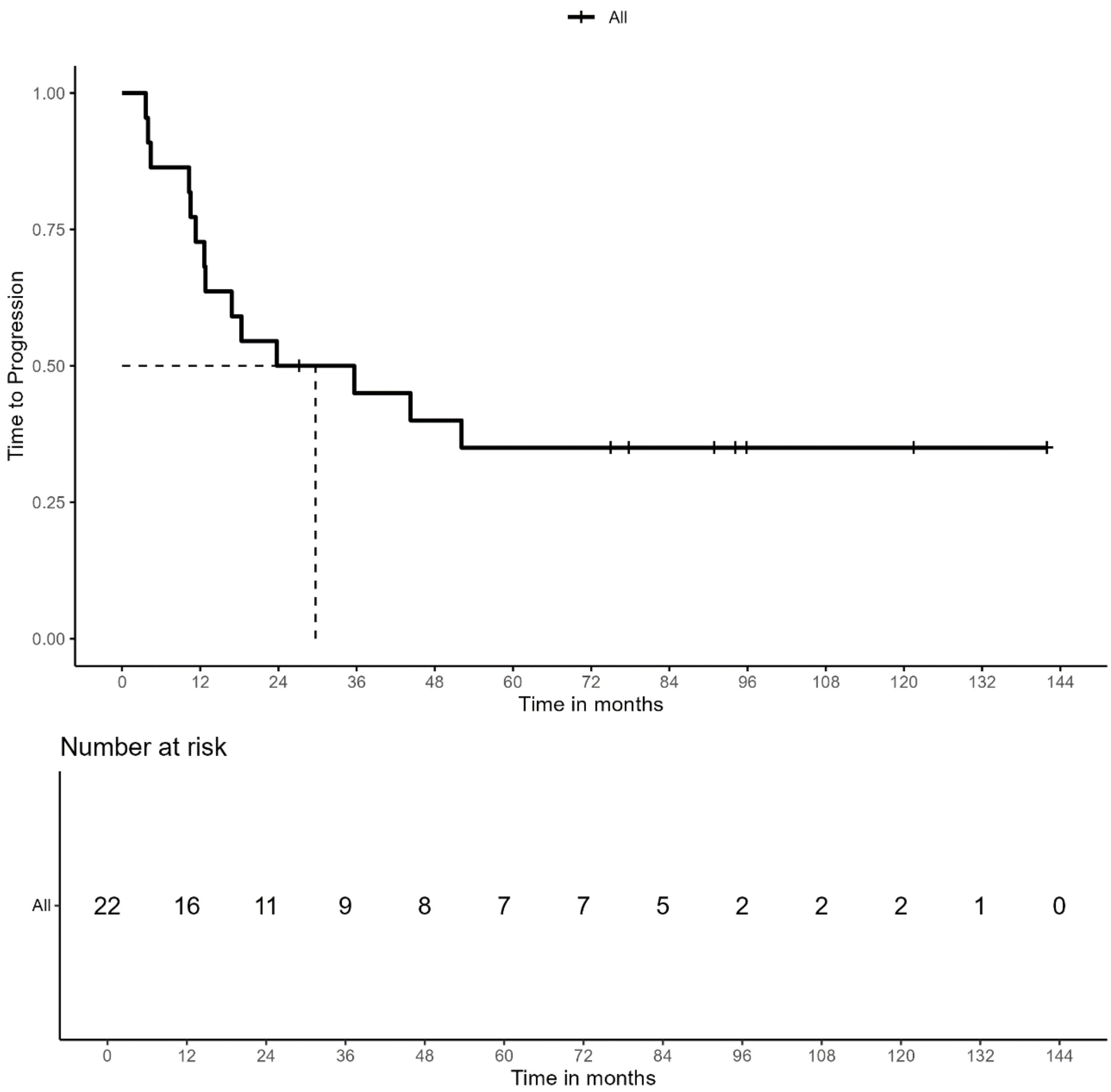

Figure S2. Time to progression (TTP) in overall biomarker-assessed-cohort.

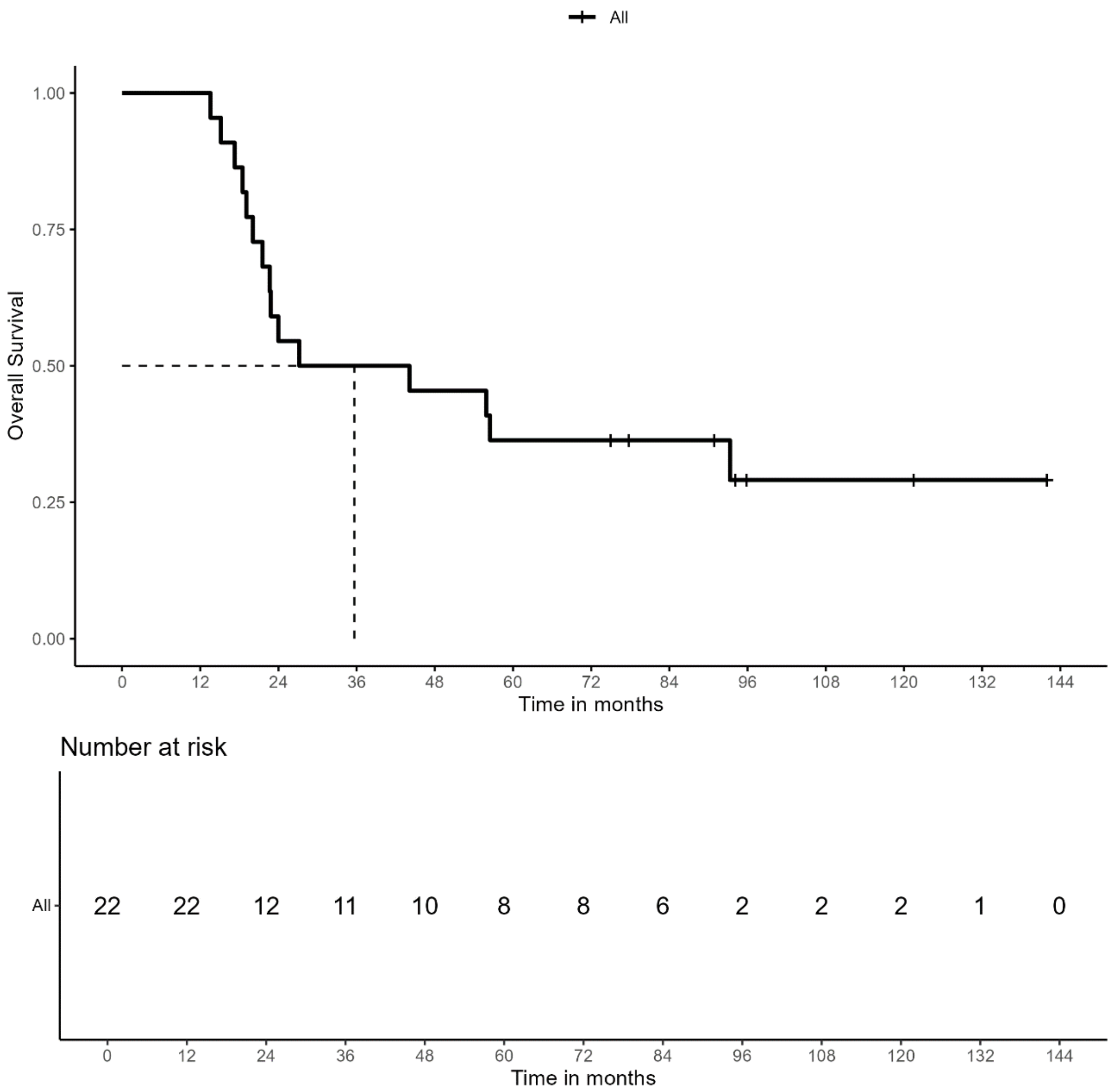

Figure S3. Overall survival (OS) in overall biomarker-assessed-cohort.

(A)

		CD24			
		0	1+	2+	3+
CD44	0	0	2	2	0
	1+	0	1	0	2
	2+	0	3	1	1
	3+	0	6	4	0

p = 0.227

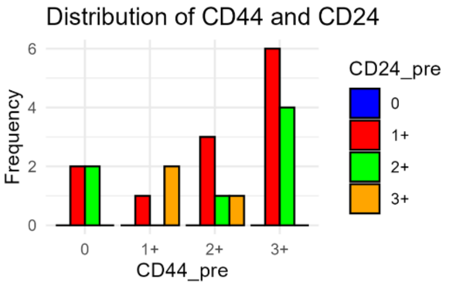

(B)

		CD24			
		0	1+	2+	3+
ALDH1	0	0	11	6	3
	1+	0	0	1	0
	2+	0	1	0	0
	3+	0	0	0	0

p = 0.714

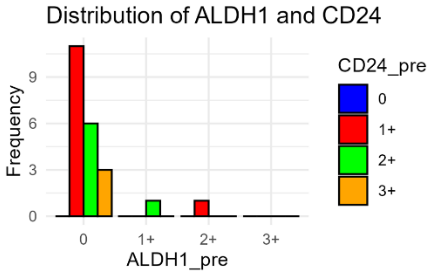

(C)

		CD44			
		0	1+	2+	3+
ALDH1	0	4	3	4	9
	1+	0	0	0	1
	2+	0	0	1	0
	3+	0	0	0	0

p = 0.805

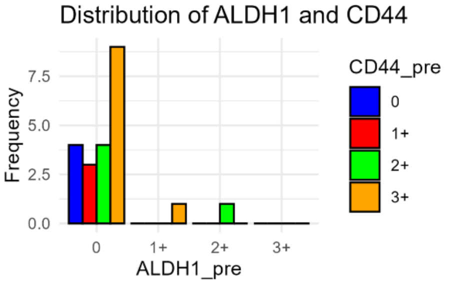

(D)

		TILs		
		0-10% A	10-40% B	40-90% C
PDL1	Negative	14	6	0
	Positive	0	1	1

*p = 0.030

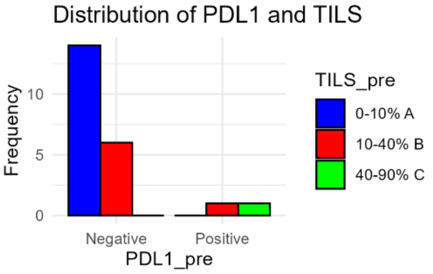

(E)

		TILs_post		
		0-10% A	10-40% B	40-90% C
PDL1_ post	Negative	9	1	0
	Positive	4	7	1

*p = 0.015

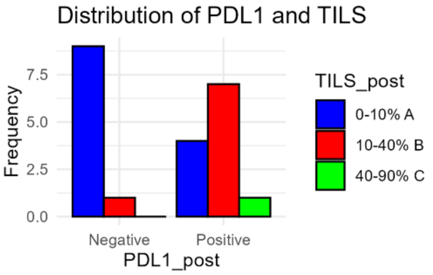

Figure S4. Association between the BCSCs and immunogenic biomarkers using a cross-tabulation, Fisher's test and a bar chart. **(A)** CD44 and CD24 basal association. **(B)** ALDH1 and CD24 basal association. **(C)** ALDH1 and CD44 basal association. **(D)** Pre-chemotherapy PDL1 and TILs association. **(E)** Post-chemotherapy PDL1 and TILs association.
